# Orange protein has a role in phytoene synthase stabilization in sweetpotato

**DOI:** 10.1038/srep33563

**Published:** 2016-09-16

**Authors:** Seyeon Park, Ho Soo Kim, Young Jun Jung, Sun Ha Kim, Chang Yoon Ji, Zhi Wang, Jae Cheol Jeong, Haeng-Soon Lee, Sang Yeol Lee, Sang-Soo Kwak

**Affiliations:** 1 Plant Systems Engineering Research Center, Korea Research Institute of Bioscience and Biotechnology (KRIBB), 125 Gwahak-ro, Daejeon 34141, Korea; 2 Department of Green Chemistry and Environmental Biotechnology, Korea University of Science and Technology (UST), 217 Gajeong-ro, Daejeon 34113, Korea; 3 Division of Applied Life Science (BK21 Plus program), Gyeongsang National University, 501 Jinjudae-ro, Jinju 52828, Korea; 4 National Institute of Ecology, 1210 Geumgang-ro, Maseo-myeon, Seocheon-gun 33657, Korea; 5 Institute of Soil and Water Conservation, Chinese Academy of Science and Ministry of Water Resources, Northwest A & F University, Shaanxi 712100, China

## Abstract

Carotenoids have essential roles in light-harvesting processes and protecting the photosynthetic machinery from photo-oxidative damage. Phytoene synthase (PSY) and Orange (Or) are key plant proteins for carotenoid biosynthesis and accumulation. We previously isolated the sweetpotato (*Ipomoea batatas*) *Or* gene (*IbOr*), which is involved in carotenoid accumulation and salt stress tolerance. The molecular mechanism underlying IbOr regulation of carotenoid accumulation was unknown. Here, we show that IbOr has an essential role in regulating IbPSY stability via its holdase chaperone activity both *in vitro* and *in vivo*. This protection results in carotenoid accumulation and abiotic stress tolerance. *IbOr* transcript levels increase in sweetpotato stem, root, and calli after exposure to heat stress. IbOr is localized in the nucleus and chloroplasts, but interacts with IbPSY only in chloroplasts. After exposure to heat stress, IbOr predominantly localizes in chloroplasts. *IbOr* overexpression in transgenic sweetpotato and *Arabidopsis* conferred enhanced tolerance to heat and oxidative stress. These results indicate that IbOr holdase chaperone activity protects IbPSY stability, which leads to carotenoid accumulation, and confers enhanced heat and oxidative stress tolerance in plants. This study provides evidence that IbOr functions as a molecular chaperone, and suggests a novel mechanism regulating carotenoid accumulation and stress tolerance in plants.

Carotenoids are essential in plants for light harvesting, photoprotection, and abscisic acid (ABA) biosynthesis^1^. Carotenoids are essential nutrients for mammalians as vitamin A precursors, antioxidants, and promoters of immune system function^2^,^3^. Due to the nutritional importance of carotenoids, metabolic engineering of carotenoid biosynthesis has been performed to enhance carotenoid contents in staple crops^1^. One of these crops, sweetpotato (*Ipomoea batatas* L. Lam.), contains abundant antioxidants, including carotenoids, anthocyanins, and vitamin C^4^. Orange-fleshed sweetpotato cultivars containing high carotenoid levels are excellent dietary sources of nutrients and antioxidants^5^. Understanding the fundamental mechanisms underlying carotenoid metabolism and accumulation is important for improving the nutritional value of sweetpotato cultivars. Several studies have performed metabolic engineering of carotenogenesis to enhance carotenoid accumulation in sweetpotato^6^,^7^,^8^,^9^.

The *Orange (Or*) gene was isolated from an orange cauliflower mutant (*Brassica oleracea* var. *botrytis*) that accumulates β-carotene in tissues normally devoid of carotenoids^10^. The *Or* gene appears to trigger the differentiation of proplastids and/or non-colored plastids into chromoplasts, which function as a metabolic sink for carotenoid accumulation^10^,^11^. *Or* encodes a DnaJ-like cysteine-rich domain-containing protein^7^,^10^. DnaJ proteins are involved in essential cellular processes such as protein folding, degradation, refolding, and homeostasis under stress conditions^12^,^13^,^14^. The DnaJ family is present in all major eukaryotic cell compartments, including the cytosol^15^, mitochondria^16^, endoplasmic reticulum^17^, and chloroplasts^18^. Chloroplasts are the site of photosynthesis in plants. Chloroplast-targeted DnaJ proteins participate in chloroplast development^19^, phototropin-mediated chloroplast movement^20^, protein import and translocation^21^, protection of photosynthetic machinery from abiotic stress^21^,^22^,^23^,^24^, and biotic stress tolerance^25^.

Phytoene synthase (PSY) is the most important regulatory enzyme in the carotenoid biosynthetic pathway. *Arabidopsis* contains only one *PSY* gene, but rice (*Oryza sativa*), maize (*Zea mays*), tomato (*Solanum lycopersicum*), and cassava (*Manihot esculenta*) contain two or more homologs^26^. Multiple *PSY* genes have tissue-specific expression and unique responses to environmental cues^26^,^27^. High light, temperature, drought, salt, ABA, photoperiod, developmental cues, and metabolite feedback affect *PSY* expression^3^. Li *et al.*^11^ reported that PSY protein level was maximally increased in transgenic potato expressing cauliflower Or, and Zhou *et al.*^28^ reported that Or was a post-transcriptional regulator of PSY. In addition, activation and translocation of PSY are regulated by post-translational effects^29^, and Or-mediated increase in PSY protein level increases PSY activity^28^. Therefore, PSY is controlled by post-transcriptional and post-translational modification, and it regulates the first committed step in carotenoid biosynthesis^3^.

Our previous studies showed that expression of the sweetpotato *Or (IbOr*) transgene in sweetpotato calli resulted in increased carotenoid levels^7^. We observed that transgenic sweetpotato calli overexpressing IbOr had higher carotenoid levels, increased antioxidant activity, and enhanced salt stress tolerance^7^. However, it was unclear how IbOr regulated carotenoid accumulation, although plant DnaJ proteins are reported as heat-shock proteins involved in abiotic stress tolerance and holdase chaperone function^30^. Here, we report the holdase chaperone function of IbOr, which regulates IbPSY stability, enhances carotenoid accumulation, and confers heat stress tolerance in sweetpotato.

## Results

### *IbOr* transcripts are induced by heat stress treatment

We reported previously that the deduced IbOr protein contains a plastid-targeting transit sequence, two transmembrane domains, and a DnaJ-like cysteine-rich zinc finger domain that includes four repeats of the CxxCxGxG motif in the C-terminal region^7^. In plants, DnaJ/Hsp40 proteins are co-chaperones that function as partners of the highly conserved Hsp70, and are required for defense against abiotic stresses such as salinity, drought, and extreme temperatures. Previous work showed that *IbOr* transcript expression was significantly induced in response to NaCl, PEG, and H_2_O_2_^7^. To identify a possible functional role for IbOr under heat stress conditions, *IbOr* expression was analyzed by quantitative RT-PCR (qRT-PCR) in heat-treated sweetpotato tissues (Supplementary Fig. S1). *IbOr* expression in stem and fibrous root was high at 3 h after heat treatment, and its expression in calli was high at 6 h after treatment. By contrast, *IbOr* expression in leaf decreased after heat treatment. To test the effect of heat treatment on IbOr protein, we purified bacterially-expressed recombinant GST:IbOr protein and evaluated its stability under heat stress conditions. GST:IbOr was stable even at 70 °C, whereas GST was aggregated at 60 °C (Supplementary Fig. S2). These results suggest that *IbOr* may play an important role in the response to heat stress in sweetpotato.

### IbOr functions as a holdase chaperone

**Figure 1:**
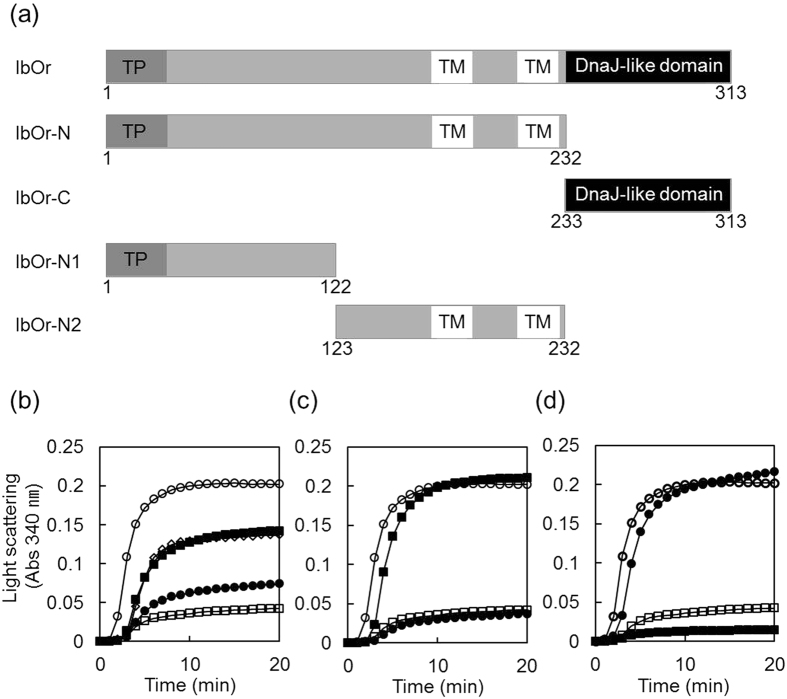
Functional analysis of IbOr as a holdase chaperone. (**a**) Schematic representation of IbOr domains and truncated forms of recombinant IbOr. TP, transit peptide; TM, transmembrane domain; DnaJ-like domain, DnaJ-like cysteine-rich zinc finger domain. (**b**) Holdase chaperone activity of IbOr in the presence of malate dehydrogenase (MDH) as determined by light scattering. Molar ratios of MDH to IbOr were 1:1 (◽), 1:0.5 (⦁), or 1:0.25 (◾). (**c**) Holdase chaperone activity of the N-terminal region (IbOr-N) and the C-terminal region (IbOr-C). Molar ratios of MDH to IbOr-N (⦁) or IbOr-C (◾) were 1:1. (**d**) Holdase chaperone activity of IbOr-N1 and IbOr-N2. Molar ratios of MDH to IbOr-N1 (⦁) or IbOr-N2 (◾) were 1:1. Thermal aggregation of 1 μM MDH was examined at 45 °C for 20 min in the presence of full-length or truncated IbOr. Reactions performed with 30 μM ovalbumin (⚪) or 1 μM AtTRX-h3 (◊) instead of IbOr were used as negative and positive controls, respectively.

Recent work reported that one of the chloroplast development-related proteins, CDF1 containing a DnaJ-like domain and three transmembrane domains, functions as holdase chaperone^30^. IbOr also contains a DnaJ-like domain and transmembrane domains, and has high thermostability. Therefore, we hypothesized that IbOr may function as a molecular chaperone. To test this hypothesis, we examined IbOr for holdase chaperone activity using malate dehydrogenase (MDH) as a substrate *in vitro*. MDH was incubated at 45 °C for 20 min with increasing amounts of recombinant full-length IbOr protein fused to GST. IbOr prevented thermal aggregation of MDH, and MDH aggregation was completely blocked at a subunit molar ratio of 1 MDH/1 IbOr (Fig. 1b). IbOr conferred greater thermotolerance activity than the positive control AtTrx-h3^31^ (Fig. 1b).

Next, we determined the IbOr region with holdase chaperone activity. We firstly generated two IbOr truncated fragments (IbOr-N and IbOr-C) (Fig. 1a). The N-terminal region of IbOr (IbOr-N) contains the transit sequence and transmembrane damains, whereas the C-terminal region of IbOr (IbOr-C) contains the DnaJ-like cysteine-rich zinc finger domain. Purified IbOr-N protein suppressed thermal aggregation of MDH, whereas IbOr-C protein had no effect (Fig. 1c). Therefore, we produced two IbOr-N truncated fragments (IbOr-N1 and IbOr-N2) (Fig. 1a). IbOr-N1 retained the transit sequence but deleted the transmembrane domains, whereas IbOr-N2 deleted the transit sequence but retained the transmembrane domains. IbOr-N2 displayed higher holdase chaperone activity than native IbOr, whereas IbOr-N1 did not show any holdase chaperone activity (Fig. 1d). Holdase chaperone activity is reported to be directly proportional with the degree of protein hydrophobicity^31^. Hydrophobicity analysis predicted that the highest hydrophobicity region in IbOr was IbOr-N2 (Supplementary Fig. S3). These results indicate that the N-terminal transmembrane domains are required for IbOr holdase chaperone activity.

### Recombinant IbOr forms a high molecular weight (HMW) protein complex

**Figure 2:**
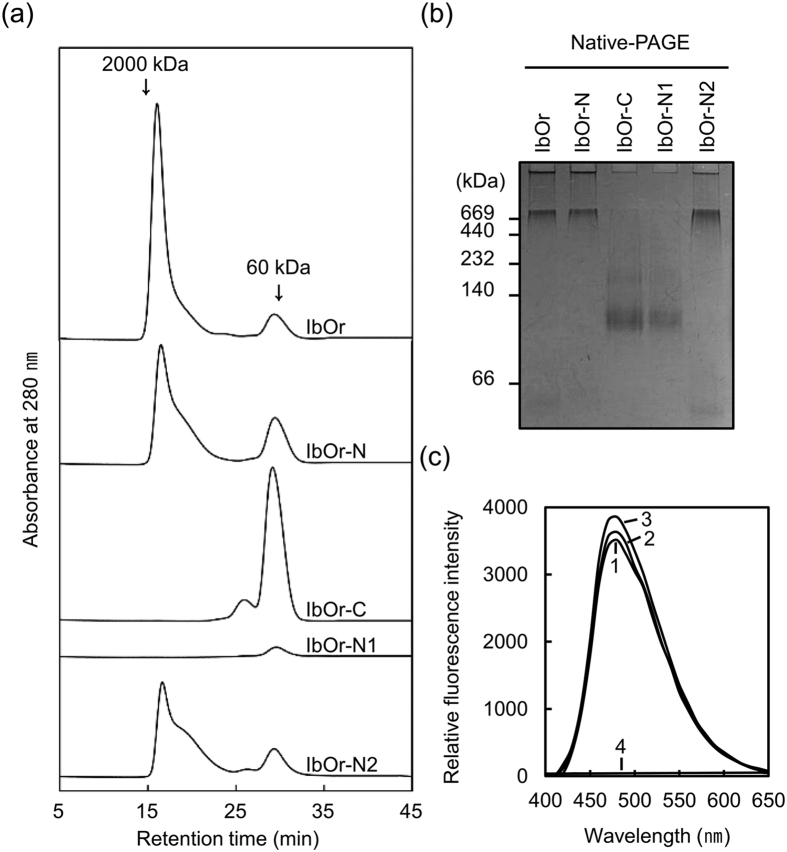
Oligomerization status of full-length IbOr and truncated fragments. (**a**) Size exclusion chromatography was performed using HPLC. Molecular masses of known standards eluted on the same column are indicated in the chromatogram. (**b**) Oligomeric status of full-length IbOr and truncated fragments was confirmed by analysis on 10% native PAGE gel followed by silver staining. (**c**) IbOr hydrophobicity was evaluated by bis-ANS binding. IbOr was incubated with bis-ANS at 25 °C for 30 min. Curve 1, 10 μM IbOr; Curve 2, 20 μM IbOr; Curve 3, 30 μM IbOr; Curve 4, 0 μM IbOr. Relative fluorescence intensities of bis-ANS were measured using an excitation wavelength of 390 nm and emission wavelengths of 400–600 nm.

HMW complex formation is a conserved feature of holdase chaperone^31^,^32^. IbOr is a heat-stable protein (Supplementary Fig. S2) that exhibits holdase chaperone function (Fig. 1b); therefore, we examined the oligomeric status of full-length protein and truncated fragments of recombinant IbOr. Size exclusion chromatography (SEC) analysis showed that full-length IbOr, IbOr-N, and IbOr-N2 primarily consisted of HMW complexes, whereas no HMW complexes were detected for IbOr-C and IbOr-N1 (Fig. 2a). Oligomeric status was confirmed using a silver-stained 10% native PAGE gel (Fig. 2b). The molecular sizes of IbOr, IbOr-N, and IbOr-N2 were too great to penetrate the 10% native polyacrylamide gel matrix, but the sizes were estimated to range up to approximately 1,000 kD. By contrast, IbOr-C and IbOr-N1 appeared to migrate as trimers. Immunoblotting analysis using GST antibody showed that all IbOr protein fragments produced a single band with the correct theoretical molecular mass (Supplementary Fig. S4). These results suggest that IbOr forms HMW complexes under normal conditions, which are homopolymers consisting of variable numbers of monomers.

We analyzed the effect of heat-shock treatment on IbOr oligomerization status. IbOr protein structure was affected *in vitro* by incubating the protein above 45 °C (Supplementary Fig. S5). As the temperature increased, the concentration of HMW complexes increased concomitantly with a decrease in the levels of oligomeric proteins. We examined IbOr hydrophobicity using the fluorescent probe 4,4′-dianilino-1,1′-binaphthyl-5,5′-disulfonic acid (bis-ANS), which binds hydrophobic regions. The fluorescence intensity of protein-bound bis-ANS increased in an IbOr concentration-dependent manner (Fig. 2c). These results suggest that IbOr holdase chaperone activity is determined by its oligomerization status.

### IbOr interacts directly with IbPSY

Zhou *et al.*^28^ recently reported that AtOr physically interacted with PSY and functioned as the major regulator of active PSY protein abundance. Therefore, we characterized sweetpotato PSY (IbPSY) and examined its interaction with IbOr. We first isolated *IbPSY* cDNA from the storage roots of orange-fleshed sweetpotato (cv. Sinhwangmi) (Accession no. JX393305). *IbPSY* had 76–96% sequence homology with several plant *PSY* genes (Supplementary Fig. S6a). To determine the subcellular localization of IbPSY, a green fluorescent protein (GFP) fusion construct of IbPSY was transiently expressed in *Nicotiana benthamiana* leaves using agroinfiltration. Epidermal cells of infiltrated leaves were examined by confocal laser scanning microscopy. The results showed that GFP fluorescence of IbPSY:GFP was detected in chloroplasts (Supplementary Fig. S6c).

**Figure 3:**
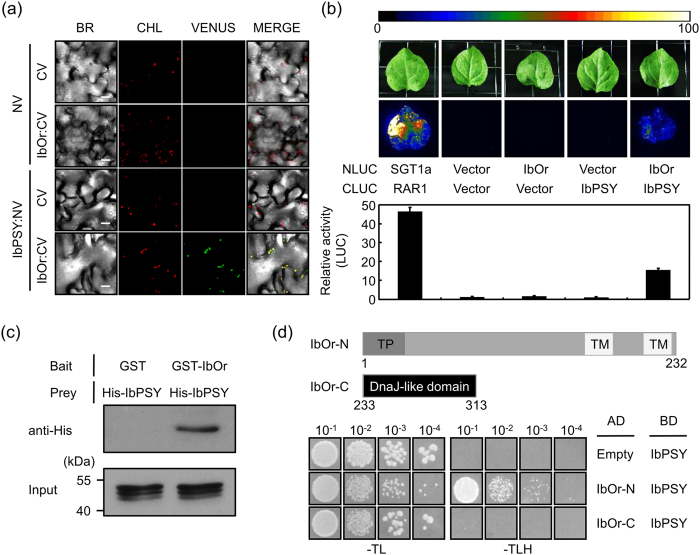
IbOr interacts with IbPSY. (**a**) Bimolecular fluorescence complementation assays for *in planta* interaction of IbOr with IbPSY in chloroplasts. *Nicotiana benthamiana* leaves were transformed by *Agrobacterium* harboring N-terminal region of Venus (NV) and C-terminal region of Venus (CV) construct pairs and observed by confocal laser scanning microscopy. BR, bright field microscopy images; CHL, chlorophyll autofluorescence; VENUS, Venus fluorescence images; MERGE, overlay of bright field, chlorophyll, and Venus images. Scale bar = 20 μm. (**b**) Luciferase complementation imaging assays for *in planta* interaction of IbOr with IbPSY. *N. benthamiana* leaves were transformed by *Agrobacterium* harboring N-terminal region of Luciferase (NLUC) and C-terminal region of Luciferase (CLUC) construct pairs. The images (top panel) and quantitative luminescence measurements (bottom panel) are shown. Results are means ± SD from three biological replicates. (**c**) Pull-down assay for *in vitro* interaction of IbOr with IbPSY. Gels containing pull-down assay products were immunoblotted with anti-His. His-IbPSY, GST (negative control), and GST-IbOr proteins are shown in indicated combinations. (**d**) Yeast two-hybrid assays for IbOr interaction with IbPSY. Schematic of IbOr domains and truncated fragments (top panel). IbOr-N or IbOr-C was fused to the activating domain (AD), and IbPSY was fused to the binding domain (BD). Yeast cells transformed with different combinations of constructs were spotted on minimal medium without Trp and Leu (−TL) and selective medium without Trp, Leu, and His (−TLH).

To determine whether IbOr and IbPSY interact, we first performed bimolecular fluorescence complementation (BiFC) assays in *N. benthamiana* leaves. The N-terminal half of Venus (improved YFP variant) was fused to IbPSY (IbPSY:NV) and the C-terminal half of Venus was fused to IbOr (IbOr:CV), and they were co-expressed in *N. benthamiana* leaf epidermal cells. The results showed strong Venus fluorescence in chloroplasts (Fig. 3a). Next, we performed luciferase (LUC) complementation imaging (LCI) assays in *N. benthamiana* leaves. LUC activity was detected by combining IbOr:NLUC with CLUC:IbPSY (Fig. 3b), which indicates that IbOr interacts with IbPSY *in planta*. This interaction was confirmed by *in vitro* pull-down assays (Fig. 3c).

Yeast two-hybrid analysis was performed to define the IbOr domain that interacts with IbPSY. A schematic diagram of the IbOr deletion constructs used in these assays is shown in Fig. 3d. The IbOr DnaJ-like domain was not required for IbPSY interaction, whereas the N-terminal region (1–232 amino acids) interacted with IbPSY (Fig. 3d). Therefore, the interaction between IbOr and IbPSY required the IbOr-N region.

### IbOr chaperone activity stabilizes IbPSY

**Figure 4:**
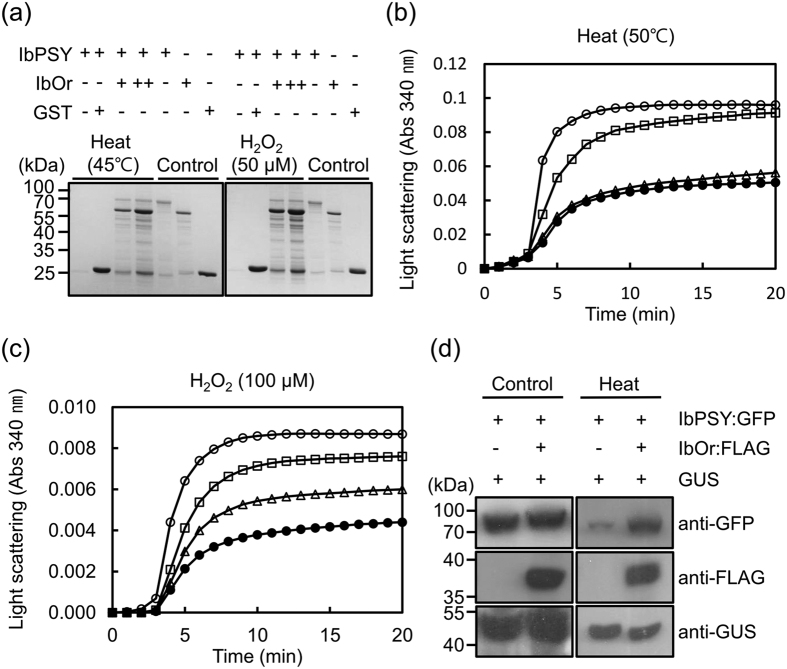
IbOr holdase chaperone activity for IbPSY. (**a**) IbOr regulates IbPSY stability under heat and oxidative stress conditions. Purified IbPSY was incubated at 45 °C for 10 min or treated with 50 μM H_2_O_2_ for 30 min with or without IbOr. IbPSY levels were detected by silver staining 12% SDS-PAGE gels. Purified IbPSY, IbOr, and GST were loaded as controls. (**b**) Thermal aggregation of IbPSY (1 μM) was examined at 50 °C for 20 min with different concentrations of IbOr. The molar ratios of IbPSY to IbOr were 1:1 (◻), 1:3 (▵), and 1:5 (⦁). (**c**) IbPSY (1 μM) aggregation in the presence of 100 μM H_2_O_2_ was examined at 30 °C for 20 min with different concentrations of IbOr. The molar ratios of IbPSY to IbOr were 1:1 (◻), 1:3 (▵), and 1:6 (⦁). For the negative control, aggregation was evaluated with 30 μM ovalbumin (○) instead of IbOr. (**d**) IbOr regulates IbPSY stability during heat stress *in planta. Nicotiana benthamiana* leaves were transiently transformed by *Agrobacterium* harboring IbPSY:GFP, IbOr:FLAG, and GUS. Transformed leaves were treated with heat stress at 37 °C for 1 h. Total protein extracts were analyzed by immunoblotting with anti-GFP, anti-FLAG, or anti-GUS antibodies. GUS protein was used as an expression and loading control.

We examined whether IbOr chaperone activity protects IbPSY from heat or oxidative stress-induced denaturation and aggregation. We treated purified recombinant GST:IbPSY protein with heat (45 °C) or oxidative (50 μM H_2_O_2_) stress in the presence or absence of purified recombinant GST:IbOr protein, and then examined the proteins on SDS-PAGE. IbPSY was aggregated under heat and oxidative stress conditions in the absence of IbOr, whereas the presence of IbOr protected IbPSY from aggregation (Fig. 4a). Next, we evaluated IbOr holdase chaperone activity using IbPSY as a substrate. IbOr prevented thermal aggregation of IbPSY in a concentration-dependent manner, and a molar ratio of 1:3 (substrate:chaperone) completely suppressed IbPSY aggregation (Fig. 4b). We tested whether IbOr prevented IbPSY aggregation induced by oxidative stress *in vitro*. At 25 °C, IbPSY treatment with 100 μM H_2_O_2_ for 20 min induced aggregation; however, the presence of IbOr suppressed IbPSY aggregation in a concentration-dependent manner (Fig. 4c). To confirm IbOr holdase chaperone activity for IbPSY *in planta*, IbOr:Flag and IbPSY:GFP fusion constructs were transiently co-expressed in *N. benthamiana* leaves by agroinfiltration. Then, these plants were subjected to heat stress at 37 °C for 1 h. The infiltrated leaves were detached and total protein extracts were prepared, which were analyzed by SDS-PAGE and immunoblotting. Under normal condition (25 °C), IbPSY levels were essentially equivalent in leaves with or without IbOr co-expression, whereas IbPSY levels were severely reduced in leaves in the absence of IbOr under heat stress conditions at 37 °C (Fig. 4d). Taken together, these results indicate that IbOr has holdase chaperone activity for IbPSY, and IbOr stabilizes IbPSY during heat stress conditions *in planta*.

### IbOr overexpression enhances abiotic stress tolerance in transgenic plants

**Figure 5:**
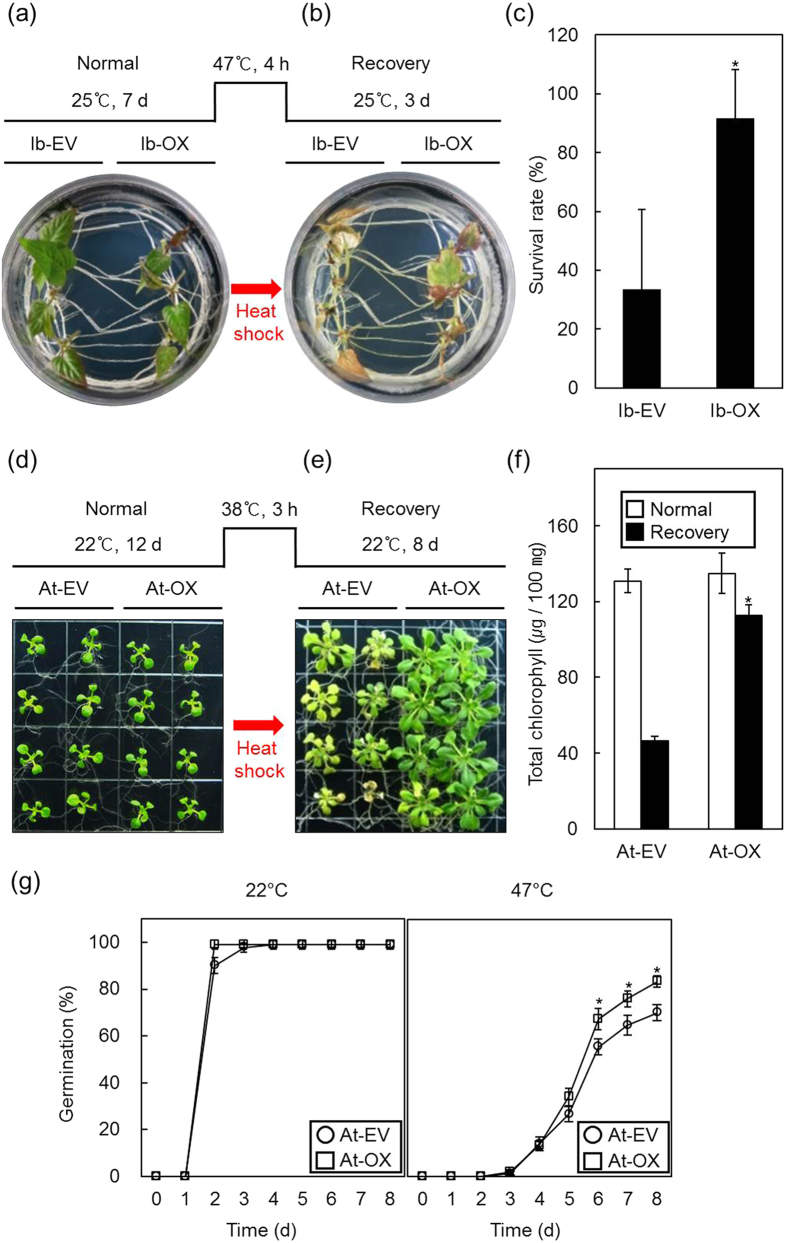
IbOr overexpression in transgenic sweetpotato plants enhances heat stress tolerance. (**a**) Phenotypes of IbOr-overexpressing (*Ib*-OX) and empty vector control (*Ib*-EV) sweetpotato transgenic plants. Seedlings were grown at 25 °C for 7 d after subculture. (**b**) Thermotolerance of *Ib*-EV and *Ib*-OX transgenic plants. A comparison of seedlings on the final day of recovery after heat shock is shown. Scheme of heat shock treatment and recovery of the seedlings is depicted (top panel). (**c**) Survival rates of *Ib*-EV and *Ib*-OX seedlings were determined after recovery. Results are means ± SD from four biological replicates. (**d**) Phenotypes of IbOr-overexpressing (*At*-OX) *Arabidopsis* plants and empty vector control (*At*-EV) *Arabidopsis* plants. Seedlings grown at 22 °C for 12 d are shown. (**e**) Thermotolerance of *At*-EV and *At*-OX transgenic plants. A comparison of seedlings on the final day of recovery after heat shock is shown. Scheme of heat shock treatment and recovery of the seedlings is depicted (top panel). (**f**) Total chlorophyll contents of *At*-EV and *At*-OX seedlings under normal temperature (NT) and after recovery. Results are means ± SD from three biological replicates. (**g**) Germination assays of *At*-EV and *At*-OX transgenic seeds under heat stress conditions. *At*-OX and *At*-EV seeds were germinated on MS agar plates with or without heat stress at 47 °C for 4 h. The germination rates were determined 1–8 d after vernalization. Results are means ± SE from three biological replicates. Asterisks indicate a significant difference between EV and OX plants at *p < 0.05 by *t*-test in (**c**,**f**,**g**).

Holdase chaperone activity confers heat stress tolerance in plants^31^,^32^. To test the physiological role of IbOr in heat-shock tolerance *in vivo*, we subjected the transformed sweetpotato plants with empty vector (*Ib*-EV) or *IbOr* overexpression construct (*Ib*-OX)^33^ to heat stress conditions. IbOr expression levels in transgenic sweetpotato were determined by anti-FLAG immunoblotting analysis (Supplementary Fig. S7). The *Ib*-EV and *Ib*-OX phenotypes were not significantly different under normal growing conditions (25 °C, Fig. 5a) and displayed similar levels of heat stress damage in response to 47 °C for 4 h. However, when the heat-stressed plants were allowed to recover at 25 °C for 3 d, the *Ib*-OX lines showed substantially superior recovery and survival than the *Ib*-EV lines (Fig. 5b,c).

To confirm IbOr-mediated improvement of heat stress tolerance in other plants, we generated transgenic *Arabidopsis* lines overexpressing FLAG-tagged IbOr. IbOr expression levels in transgenic *Arabidopsis* were determined by anti-FLAG immunoblotting analysis (Supplementary Fig. S7). The phenotypes of overexpression lines (*At*-OX) and empty vector control lines (*At*-EV) were not significantly different under normal growing conditions (22 °C, Fig. 5d), and were similarly damaged by heat stress at 38 °C for 3 h. When the heat-stressed plants were allowed to recover at 22 °C for 8 d, the *At-*OX lines displayed enhanced heat-shock tolerance and superior recovery of growth and normal chlorophyll content than the *At*-EV lines (Fig. 5e,f). When *At*-OX and *At*-EV seeds were subjected to heat stress conditions, the germination rate of *At*-OX seeds was significantly higher than that of At-EV seeds (Fig. 5g).

**Figure 6:**
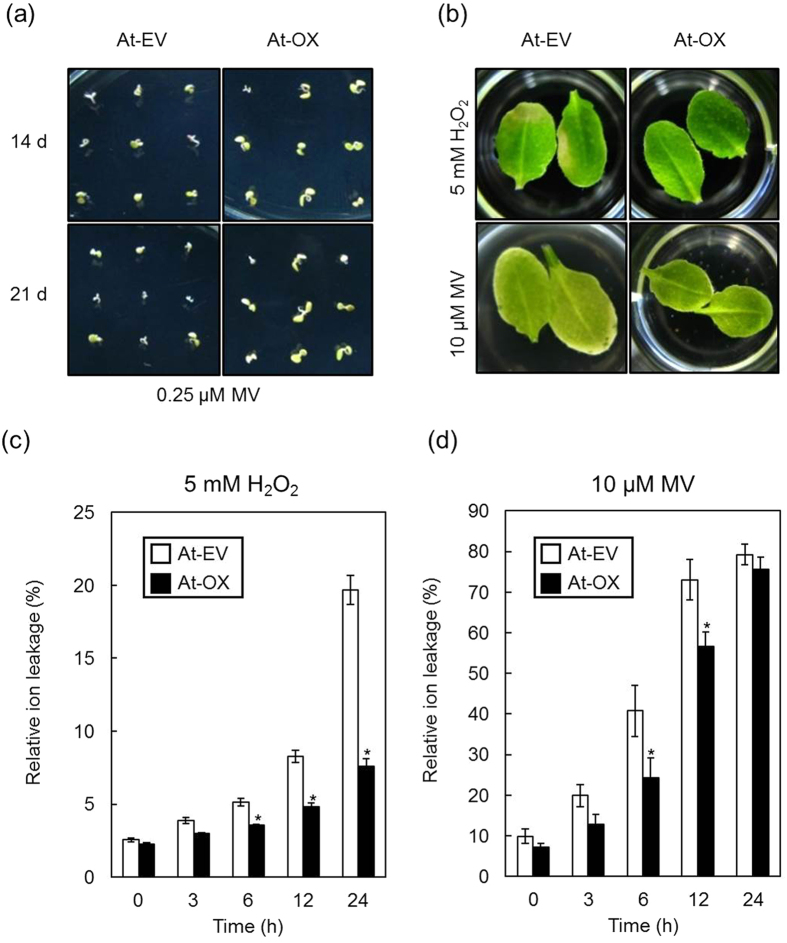
IbOr overexpression enhances oxidative stress tolerance. (**a**) Visual comparison of seed germination and seedling growth for T_3_ *At*-EV and *At*-OX (IbOr overexpression) seeds after 14 d (top panel) and 21 d (bottom panel) in the presence of 0.25 μM methyl viologen. (**b**) Progression during 24 h of stress-induced damage caused by 5 mM H_2_O_2_ or 10 μM methyl viologen treatment of *At*-EV and *At*-OX rosette leaves. (**c**,**d**) Relative ion leakage from *At*-EV and *At*-OX rosette leaves after treatment with H_2_O_2_ (**c**) or methyl viologen (**d**) for 24 h. Results are the means ± SD from three biological replicates. Asterisks indicate a significant difference between EV and OX plants at *p < 0.05 by *t*-test.

Oxidative stress was reported as a key factor that exacerbated the detrimental effects of heat stress in plants^34^. Therefore, we investigated the physiological responses of transgenic *At*-EV and *At*-OX under oxidative stress conditions. *At*-OX lines displayed enhanced tolerance to methyl viologen (MV, an inducer of oxidative stress) treatment during germination and seedling growth (Fig. 6a). When detached rosette leaves of *At*-EV and *At*-OX were treated with H_2_O_2_ or MV, the *At*-OX lines exhibited fewer damage symptoms (Fig. 6b–d). Taken together, these results suggest that IbOr has a crucial role in plant protection from heat and oxidative stress.

## Discussion

**Figure 7:**
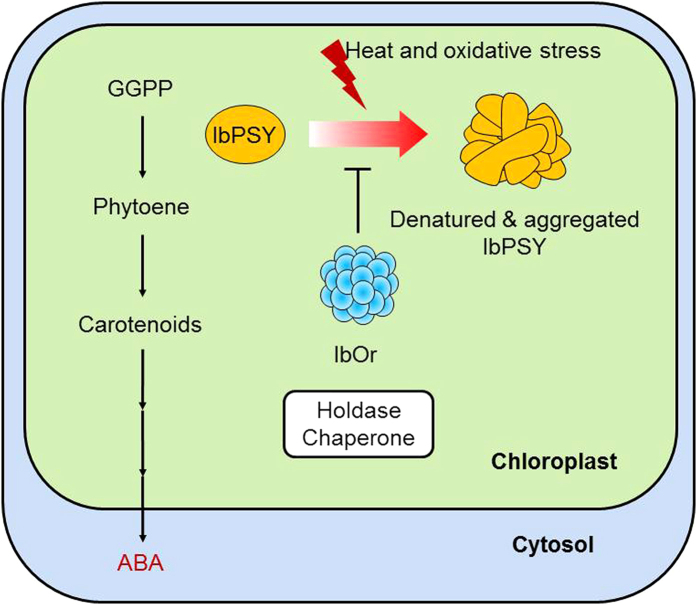
Representative model of IbOr function in response to stress. When plants are exposed to oxidative and heat stresses, IbOr holdase chaperone activity is required to prevent IbPSY aggregation. IbOr-mediated protection of IbPSY leads to carotenoid accumulation and stress tolerance.

IbOr is a key protein involved in carotenoid accumulation and environmental stress tolerance in sweetpotato^7^,^33^, but the molecular mechanism of IbOr function was previously unknown. Here, we report a novel molecular function for IbOr in stabilizing chloroplastic IbPSY via its holdase chaperone activity. Further, we demonstrate that IbOr enhances abiotic stress tolerance in transgenic plants. Our results provide new insights into the molecular mechanism of Orange protein function, which post-translationally regulates IbPSY and thereby affects carotenoid biosynthesis and accumulation. Our results are summarized in the model shown in Fig. 7.

In *Arabidopsis*, AtOr directly interacts with AtPSY in plastids^28^. PSY levels strongly increase in *AtOr*-overexpressing lines and dramatically decline in *ator* and *ator-like* double mutants, without any transcriptional change in *PSY* expression^28^. However, the mechanism of Or-mediated PSY regulation remained undetermined. PSY is reported to regulate carotenoid biosynthesis under abiotic stress conditions^26^,^27^, but IbPSY aggregates under heat and oxidative stresses (Fig. 4a). This result suggested that IbPSY may receive protection from partner protein(s) during abiotic stress conditions. We found that IbOr directly interacted with IbPSY in the chloroplast, similar to the interaction between AtOr and AtPSY. Our results also determined that IbPSY is protected by IbOr holdase chaperone activity under heat and oxidative stress conditions. PSY stability also was enhanced by *Or* transgene expression in cold-storage potato tuber^11^. The combined evidence indicates that IbOr has a role in post-translational regulation of IbPSY, and thereby controls carotenoid biosynthesis and accumulation and abiotic stress responses.

Plant Or proteins contain an N-terminal unknown region, transmembrane domains, and a C-terminal DnaJ-like domain. These domains are highly conserved among plant species^7^,^10^. CDF1 protein contains a DnaJ-like domain and three transmembrane domains^30^. Both CDF1 and IbOr have holdase chaperone activity. CDF1 required both the DnaJ-like domain and the transmembrane domains for holdase chaperone function, whereas IbOr only required the transmembrane domains that exhibited the strongest holdase chaperone activity. In *Arabidopsis*, AtPSY interacts with the AtOr N-terminal unknown region^28^. In sweetpotato, IbPSY interacted with the IbOr-N fragment (1–232 amino acids), which contains the N-terminal unknown region (30–153 amino acids) and the transmembrane domains (154–232 amino acids). Both the IbOr N-terminal unknown region and the IbOr C-terminal DnaJ-like domain have been reported to be involved in protein-protein interactions, suggesting that Or may be multi-functional protein^28^,^35^. The Orange protein N-terminal region interacts with PSY in the chloroplast and is involved in regulating the homeostasis of photosynthesis and carotenoid biosynthesis^28^, whereas the C-terminal DnaJ-like domain interacts with eRF1–2 in the nucleus and controls leaf petiole elongation^35^. IbOr also is mainly localized in the nucleus (Supplementary Fig. S8, top panel), and IbOr localization prominently changes to the chloroplast in response to heat stress (Supplementary Fig. S8, bottom panel). This suggests that IbOr might translocate to the chloroplast during heat stress to protect IbPSY from heat stress-induced aggregation. Subcellular protein translocation in response to oxidative stress condition has been reported in plants^36^. The potential subcellular translocation of IbOr in response to environmental stress conditions is consistent with Or function. These combined results indicate that Or is a multi-functional protein involved in plant growth, development, and abiotic stress responses.

Transcript levels of several plant *DnaJ* genes targeted to the chloroplast are induced by abiotic stresses^23^. We reported previously that *IbOr* expression also responds to abiotic stresses including salt, drought, and oxidative stress^7^. In this study, we found that *IbOr* transcript expression was induced by heat stress in sweetpotato stem, fibrous root, and calli, but not in leaves. Zhou *et al.*^28^ recently reported that *AtOr* transcript levels were greatly reduced in *psy* co-suppressed plants. Similarly, heat stress may severely suppress *IbPSY* expression in leaves and lead to reduced *IbOr* transcript levels. In heat-stressed leaves, IbOr translocated to the chloroplast to protect IbPSY (Supplementary Fig. S8). These results indicate that *IbOr* displays tissue-specific responses to heat stress.

DnaJ proteins belong to a large protein family that is characterized by different subcellular localizations^37^. However, the majority of DnaJ proteins (including Or) are localized in the chloroplast^38^. Chloroplast-targeted DnaJ proteins have important roles in photosynthesis because they are involved in maintaining PSII function^23^, protecting Rubisco activity^24^, chloroplast development^21^,^30^, PSI accumulation^39^, and optimizing photosynthetic reactions^22^. Carotenoids are essential for photosynthesis, and PSY catalyzes the rate-limiting step of carotenoid biosynthesis^3^. Because Or regulates PSY, Or is involved in photosynthesis via regulation of carotenoid biosynthesis. The *Arabidopsis ator* and *ator-like* double mutants exhibited a pale green phenotype with reduced carotenoid contents due to the loss of chlorophyll and disruption of carotenoid homeostasis^28^. Transgenic sweetpotato and *Arabidopsis* plants overexpressing *IbOr* displayed enhanced heat stress tolerance and higher chlorophyll contents than those of control plants transformed with empty vector. Stress tolerance of *IbOr*-OX plants is likely caused by enhanced stability of photosynthetic proteins and controlled homeostasis of chlorophyll and carotenoids. Photosynthesis is sensitive to heat stress. The protection of photosynthetic enzymes and cofactors protects photosynthetic reactions and accessory pathways, and thereby enhances stress tolerance^24^. *IbOr*-OX *Arabidopsis* plants also displayed enhanced oxidative stress tolerance. Recent reports show that *IbOr* overexpression enhances abiotic stress tolerance in sweetpotato calli^7^, alfalfa^40^, and potato^41^. These results suggest that IbOr has a crucial role in maintenance of photosynthesis, which thereby confers stress tolerance.

Conclusively, our results indicated that IbOr plays a role in stabilization of IbPSY in response to heat and oxidative stresses. In addition, holdase chaperone function of IbOr is involved in carotenoid biosynthesis by protection of IbPSY and tolerance to environmental stress in plant. This work will provide a new strategy to develop plants with enriched carotenoids contents and enhanced environmental stress tolerance.

## Methods

### Plant materials, growth conditions, and stress treatments

Orange-fleshed sweetpotato plants [*Ipomoea batatas* (L.) Lam. cv. Sinhwangmi], sweetpotato transgenic lines overexpressing empty vector (*Ib*-EV) and IbOr (*Ib*-OX)^33^, *Arabidopsis thaliana* (ecotype Columbia-0), and *Nicotiana benthamiana* were used in this study. Orange-fleshed sweetpotato plants were obtained from the Bioenergy Crop Research Center, National Institute of Crop Science, Rural Development Administration, Korea. Plants were cultivated in plastic pots filled with soil in a growth room at 25 or 22 °C under 16 h light/8 h dark photocycles. Three-week-old sweetpotato plants were used for *IbOr* expression analysis under heat stress conditions. Sweetpotato calli were induced from storage roots and cultured on MS^42^ medium supplemented with 1 mg/L 2,4-dichlorophenoxyacetic acid (2,4-D), 3% sucrose, and 0.4% Gelrite (MS1D). Calli were proliferated on MS1D media with 21 d subculture intervals and incubated at 25 °C in the dark. Sweetpotato calli 10 d after subculture were used for *IbOr* expression analysis under heat stress conditions. Sweetpotato transgenic lines, *Ib*-EV and *Ib*-OX, were cultivated on MS plates with 21 d subculture intervals and incubated in a growth room at 25 °C under 16 h light/8 h dark photocycles. To test heat-shock tolerance, *Ib*-EV and *Ib*-OX were grown on MS plates for 1 week after subculture, subjected to heat treatment at 47 °C for 4 h, and then returned to 25 °C for recovery. The plants’ ability to recover growth following heat shock was then analyzed.

*Arabidopsis* transgenic lines overexpressing empty vector (*At*-EV) or IbOr (*At*-OX) were generated as follows. The pGWB11 or pGWB11-*IbOr-Wt* plant expression vector^7^ was transformed into *Agrobacterium tumefaciens* GV3101 and introduced into *Arabidopsis* using the flower-dipping method^43^. IbOr protein expression was evaluated by immunoblotting analysis. For the *Arabidopsis* heat-shock tolerance experiment, *At*-EV and *At*-OX were grown on MS plates for 12 d at 22 °C under 16 h light/8 h dark photocycles, heat treated at 38 °C for 3 h, and then returned to 22 °C for recovery. The plants’ ability to recover following heat shock was then analyzed. For the heat-shock tolerance test of transgenic seed germination, stratified (3 d in the dark at 4 °C) T_3_ lines of *At*-EV and *At*-OX seeds were subjected to 47 °C for 4 h (or no heat treatment for control), sown on MS plates, and incubated for 8 d at 22 °C under 16 h light/8 h dark photocycles. Germination was assessed every 24 h and defined as emergence of the radicle. For the oxidative stress resistance experiment, *At*-EV and *At*-OX were germinated and grown for 21 d at 22 °C under 16 h light/8 h dark photocycles on MS plates containing 0.25 μM methyl viologen, and then seedling phenotypes were analyzed. All values are averages of at least three independent measurements.

### Determination of holdase chaperone activity

*In vitro* holdase chaperone activity was evaluated using MDH and GST:IbPSY as substrates. The substrates were incubated in 50 mM HEPES-KOH (pH 8.0) buffer at 45 or 50 °C or 50 or 100 μM H_2_O_2_ with various concentrations of GST:IbOr or IbOr truncated fragments. Substrate stability was determined by SDS-PAGE, and substrate aggregation was determined by monitoring the turbidity (light scattering) at A_340_ as described previously^31^. *In planta* holdase chaperone activity was evaluated using IbPSY:GFP as substrate. Three-week-old *N. benthamiana* plants were used for *Agrobacterium*-mediated transient expression; pMDC83*-IbPSY*, pCAMBIA1300-multi-*GUS*, and pGWB11-*IbOr* or pGWB11 (EV) were transformed into *Agrobacterium tumefaciens* GV3101, and *Agrobacterium*-mediated transient expression was performed. Three days after infiltration, *N. benthamiana* plants were subjected to 38 °C for 1 h, and then total proteins were extracted. Substrate stability was determined by immunoblotting with anti-GFP, anti-FLAG, and anti-GUS.

### Size exclusion chromatography, polyacrylamide gel electrophoresis (PAGE), and immunoblot analysis

SEC was performed at 25 °C using HPLC (Dionex, Sunnyvale, CA USA) and a TSK G4000SWXL column equilibrated with 50 mM HEPES-KOH (pH 8.0) buffer containing 100 mM NaCl as described previously^31^. SDS-PAGE, native PAGE, and immunoblot analysis were performed as described previously^32^.

### Hydrophobicity analysis

IbOr hydrophobicity was determined spectrophotometrically using the SFM25 spectrofluorometer (Kontron, Basel, Switzerland). The binding of bis-ANS was measured in the presence of increasing IbOr concentrations, which revealed exposure of the IbOr hydrophobic domain^44^.

### Laser scanning confocal microscopy

Constructs were introduced into *Agrobacterium tumefaciens* EHA105 for *Agrobacterium*-mediated transient expression. Three days after infiltration, *N. benthamiana* plants were treated with 38 °C for 1 h (or no treatment for control), and then leaves were cut off into small squares. The cut leaves were fixed and stained with DAPI (to label nuclei) as described previously^45^,^46^. The samples were examined for fluorescent protein expression by confocal microscopy as described previously^46^.

### Bimolecular fluorescence complementation (BiFC) assay

Constructs were transformed into *Agrobacterium tumefaciens* EHA105, and *Agrobacterium*-mediated transient expression was performed. Three days after infiltration, *N. benthamiana* leaves were cut off into small squares. The samples were examined for Venus fluorescence by confocal microscopy as described previously^46^.

Detailed procedures of cloning and preparation of recombinant proteins, oligomerization status analysis, thermostability test, qRT-PCR analysis, firefly luciferase complementation imaging assay, pull-down assay, yeast two-hybrid assay, total chlorophyll content measurement, and ion leakage analysis are described in Supplementary Information.

## Additional Information

**How to cite this article**: Park, S. *et al.* Orange protein has a role in phytoene synthase stabilization in sweetpotato. *Sci. Rep.* **6**, 33563; doi: 10.1038/srep33563 (2016).

## Supplementary Material

Supplementary Information

## References

[b1] Giuliano G. Plant carotenoids: genomics meets multi-gene engineering. Current Opinion in Plant Biology 19, 111–117 (2014). 24912125 10.1016/j.pbi.2014.05.006

[b2] Hughes D. A. Effects of carotenoids on human immune function. Proceedings of the Nutrition Society 58, 713–718 (1999). 10604207 10.1017/s0029665199000932

[b3] Cazzonelli C. I. & Pogson B. J. Source to sink: regulation of carotenoid biosynthesis in plants. Trends in Plant Science 15, 266–274 (2010). 20303820 10.1016/j.tplants.2010.02.003

[b4] Teow C. C., Truong V. D., McFeeters R. F., Thompson R. L., Pecota K. V. & Yencho G. C. Antioxidant activities, phenolic and β-carotene contents of sweet potato genotypes with varying flesh colours. Food Chemistry 103, 829–838 (2007).

[b5] Van Jaarsveld P. J. *et al.* β -Carotene-rich orange-fleshed sweet potato improves the vitamin A status of primary school children assessed with the modified-relative-dose-response test. The American Journal of Clinical Nutrition 81, 1080–1087 (2005). 15883432 10.1093/ajcn/81.5.1080

[b6] Kim S. H., Ahn Y. O., Ahn M. J., Lee H. S. & Kwak S. S. Down-regulation of β-carotene hydroxylase increases β-carotene and total carotenoids enhancing salt stress tolerance in transgenic cultured cells of sweetpotato. Phytochemistry 74, 69–78 (2012). 22154923 10.1016/j.phytochem.2011.11.003

[b7] Kim S. H. *et al.* Cloning and characterization of an *Orange* gene that increases carotenoid accumulation and salt stress tolerance in transgenic sweetpotato cultures. Plant Physiology and Biochemistry 70, 445–454 (2013). 23835362 10.1016/j.plaphy.2013.06.011

[b8] Kim S. H. *et al.* Downregulation of the lycopene *ϵ*-cyclase gene increases carotenoid synthesis via the *β*-branch-specific pathway and enhances salt-stress tolerance in sweetpotato transgenic calli. Physiologia Plantarum 147, 432–442 (2013). 22938023 10.1111/j.1399-3054.2012.01688.x

[b9] Kim S. H. *et al.* Down-regulation of sweetpotato lycopene β-cyclase gene enhances tolerance to abiotic stress in transgenic calli. Molecular Biology Reports 41, 8137–8148 (2014). 25213547 10.1007/s11033-014-3714-4

[b10] Lu S. *et al.* The cauliflower *Or* gene encodes a DnaJ cysteine-rich domain-containing protein that mediates high levels of β-carotene accumulation. Plant Cell 18, 3594–3605 (2006). 17172359 10.1105/tpc.106.046417 PMC1785402

[b11] Li L. *et al.* The Or gene enhances carotenoid accumulation and stability during post-harvest storage of potato tubers. Molecular Plant 5, 339–352 (2012). 22155949 10.1093/mp/ssr099

[b12] Wang W., Vinocur B., Shoseyov O. & Altman A. Role of plant heat-shock proteins and molecular chaperones in the abiotic stress response. Trends in Plant Science 9, 244–252 (2004). 15130550 10.1016/j.tplants.2004.03.006

[b13] Hennessy F., Nicoll W. S., Zimmermann R., Cheetham M. E. & Blatch G. L. Not all J domains are created equal: implications for the specificity of Hsp40-Hsp70 interactions. Protein Science 14, 1697–1709 (2005). 15987899 10.1110/ps.051406805 PMC2253343

[b14] Craig E. A., Huang P., Aron R. & Andrew A. The Diverse Roles of J-proteins, the Obligate Hsp70 Co-chaperone. In Reviews of Physiology, Biochemistry and Pharmacology (eds Amara S. G., Bamberg E., Grinstein S., Hebert S. C., Jahn R., Lederer W. J., Schweiger M.), pp. 1–21. Springer, Berlin (2006). 10.1007/s10254-005-0001-0 16634144

[b15] Thomas J. G. & Baneyx F. Protein folding in the cytoplasm of Escherichia coli: requirements for the DnaK-DnaJ-GrpE and GroEL-GroES molecular chaperone machines. Molecular Microbiology 21, 1185–1196 (1996). 8898387 10.1046/j.1365-2958.1996.651436.x

[b16] Voos W. & Rottgers K. Molecular chaperones as essential mediators of mitochondrial biogenesis. Biochimica et Biophysica Acta 1592, 51–62 (2002). 12191768 10.1016/s0167-4889(02)00264-1

[b17] Nicoll W. S., Boshoff A., Ludewig M. H., Hennessy F., Jung M. & Blatch G. L. Approaches to the isolation and characterization of molecular chaperones. Protein Expression and Purification 46, 1–15 (2006). 16199180 10.1016/j.pep.2005.08.005

[b18] Orme W., Walker A. R., Gupta R. & Gray J. C. A novel plastid-targeted J-domain protein in *Arabidopsis thaliana*. Plant Molecular Biology 46, 615–626 (2001). 11516154 10.1023/a:1010665702621

[b19] Albrecht V., Ingenfeld A. & Apel K. *Snowy cotyledon 2*: the identification of a zinc finger domain protein essential for chloroplast development in cotyledons but not in true leaves. Plant Molecular Biology 66, 599–608 (2008). 18209955 10.1007/s11103-008-9291-y

[b20] Suetsugu N., Kagawa T. & Wada M. An auxilin-like J-domain protein, JAC1, regulates phototropin-mediated chloroplast movement in Arabidopsis. Plant Physiology 139, 151–162 (2005). 16113208 10.1104/pp.105.067371 PMC1203365

[b21] Reinbothe S., Gray J., Rustgi S., Von W. D. & Reinbothe C. Cell growth defect factor 1 is crucial for the plastid import of NADPH:protochlorophyllide oxidoreductase A in *Arabidopsis thaliana*. Proceedings of the National Academy of Sciences USA 112, 5838–5843 (2015). 10.1073/pnas.1506339112 PMC4426407 25901327

[b22] Chen K. M. *et al.* Small chloroplast-targeted DnaJ proteins are involved in optimization of photosynthetic reactions in *Arabidopsis thaliana*. BMC Plant Biology 10, 43 (2010). 20205940 10.1186/1471-2229-10-43 PMC2844072

[b23] Kong F. *et al.* A chloroplast-targeted DnaJ protein contributes to maintenance of photosystem II under chilling stress. Journal of Experimental Botany 65, 143–158 (2014). 24227338 10.1093/jxb/ert357 PMC3883286

[b24] Wang G. *et al.* A tomato chloroplast-targeted DnaJ protein protects Rubisco activity under heat stress. Journal of Experimental Botany 66, 3027–3040 (2015). 25801077 10.1093/jxb/erv102

[b25] Wang G. *et al.* Overexpression of tomato chloroplast-targeted DnaJ protein enhances tolerance to drought stress and resistance to *Pseudomonas solanacearum* in transgenic tobacco. Plant Physiology and Biochemistry 82, 95–104 (2014). 24929777 10.1016/j.plaphy.2014.05.011

[b26] Welsch R., Wüst F., Bär C., Al-Babili S. & Beyer P. A third phytoene synthase is devoted to abiotic stress-induced abscisic acid formation in rice and defines functional diversification of phytoene synthase genes. Plant Physiology 147, 367–380 (2008). 18326788 10.1104/pp.108.117028 PMC2330301

[b27] Li F., Vallabhaneni R., Yu J., Rocheford T. & Wurtzel E. T. The maize phytoene synthase gene family: overlapping roles for carotenogenesis in endosperm, photomorphogenesis, and thermal stress tolerance. Plant Physiology 147, 1334–1346 (2008). 18508954 10.1104/pp.108.122119 PMC2442542

[b28] Zhou X. *et al.* *Arabidopsis* OR proteins are the major posttranscriptional regulators of phytoene synthase in controlling carotenoid biosynthesis. Proceedings of the National Academy of Sciences USA 112, 3558–3563 (2015). 10.1073/pnas.1420831112 PMC4371912 25675505

[b29] Welsch R., Beyer P., Hugueney P., Kleinig H. & Von L. J. Regulation and activation of phytoene synthase, a key enzyme in carotenoid biosynthesis, during photomorphogenesis. Planta 211, 846–854 (2000). 11144270 10.1007/s004250000352

[b30] Lee J. Y. *et al.* Cell growth defect factor1/CHAPERONE-LIKE PROTEIN OF POR1 plays a role in stabilization of light-dependent protochlorophyllide oxidoreductase in *Nicotiana benthamiana* and *Arabidopsis*. Plant Cell 25, 3944–3960 (2013). 24151298 10.1105/tpc.113.111096 PMC3877821

[b31] Park S. K. *et al.* Heat-shock and redox-dependent functional switching of an h-type arabidopsis thioredoxin from a disulfide reductase to a molecular chaperone. Plant Physiology 150, 552–561 (2009). 19339505 10.1104/pp.109.135426 PMC2689952

[b32] Lee J. R. *et al.* Heat-shock dependent oligomeric status alters the function of a plant-specific thioredoxin-like protein, AtTDX. Proceedings of the National Academy of Sciences USA 106, 5978–5983 (2009). 10.1073/pnas.0811231106 PMC2667072 19293385

[b33] Park S. C. *et al.* Enhanced accumulation of carotenoids in sweetpotato plants overexpressing *IbOr-Ins* gene in purple-fleshed sweetpotato cultivar. Plant Physiology and Biochemistry 86, 82–90 (2015). 25438140 10.1016/j.plaphy.2014.11.017

[b34] Vacca R. A. *et al.* Production of reactive oxygen species, alteration of cytosolic ascorbate peroxidase, and impairment of mitochondrial metabolism are early events in heat shock-induced programmed cell death in tobacco Bright-Yellow 2 cells. Plant Physiology 134, 1100–1112 (2004). 15020761 10.1104/pp.103.035956 PMC389934

[b35] Zhou X. *et al.* The cauliflower *Orange* gene enhances petiole elongation by suppressing expression of *eukaryotic release factor 1*. New Phytologist 190, 89–100 (2011). 21175633 10.1111/j.1469-8137.2010.03578.x

[b36] Inze A. *et al.* A subcellular localization compendium of hydrogen peroxide-induced proteins. Plant, Cell and Environment 35, 308–320 (2012). 10.1111/j.1365-3040.2011.02323.x 21443605

[b37] Miernyk J. A. The J-domain proteins of *Arabidopsis thaliana*: an unexpectedly large and diverse family of chaperones. Cell Stress Chaperon 6, 209 (2001). 10.1379/1466-1268(2001)006<0209:tjdpoa>2.0.co;2 PMC434402 11599562

[b38] Chiu C. C., Chen L. J., Su P. H. & Li H. M. Evolution of chloroplast J proteins. PloS ONE 8, e70384 (2013). 23894646 10.1371/journal.pone.0070384 PMC3720927

[b39] Fristedt R., Williams-Carrier R., Merchant S. S. & Barkan A. A Thylakoid Membrane Protein Harboring a DnaJ-type Zinc Finger Domain Is Required for Photosystem I Accumulation in Plants. Journal of Biological Chemistry 289, 30657–30667 (2014). 25228689 10.1074/jbc.M114.587758 PMC4215244

[b40] Wang Z. *et al.* Transgenic alfalfa plants expressing the sweetpotato *orange* gene exhibit enhanced abiotic stress tolerance. PLoS ONE 10, e0126050 (2015). 25946429 10.1371/journal.pone.0126050 PMC4422619

[b41] Goo Y. M. *et al.* Overexpression of the sweet potato *IbOr* gene results in the increased accumulation of carotenoid and confers tolerance to environmental stresses in transgenic potato. Comptes Rendus Biologies 338, 12–20 (2015). 25528672 10.1016/j.crvi.2014.10.006

[b42] Murashige T. & Skoog F. A revised medium for rapid growth and bio assays with tobacco tissue cultures. Physiologia Plantarum 15, 473–497 (1962).

[b43] Clough S. Floral Dip: Agrobacterium-Mediated Germ Line Transformation. In Transgenic Plants: Methods and Protocols (eds Peña L.), pp. 91–101. Humana Press, Totowa (2004). 10.1385/1-59259-827-7:091 15310915

[b44] Sharma K. K., Kaur H., Kumar G. S. & Kester K. Interaction of 1,1′-bi(4-anilino)naphthalene-5,5′-disulfonic acid with α-crystallin. Journal of Biological Chemistry 273, 8965–8970 (1998). 9535881 10.1074/jbc.273.15.8965

[b45] Cheng Z. *et al.* The bHLH transcription factor MYC3 interacts with the jasmonate ZIM-domain proteins to mediate jasmonate response in *Arabidopsis*. Molecular Plant 4, 279–288 (2011). 21242320 10.1093/mp/ssq073

[b46] Poornima Priyadarshini C. G., Ambika M. V., Tippeswamy R. & Savithri H. S. Functional characterization of coat protein and V2 involved in cell to cell movement of *Cotton Leaf Curl Kokhran Virus-Dabawali*. PLoS ONE 6, e26929 (2011). 22110597 10.1371/journal.pone.0026929 PMC3217939

