# Supplementary material for: Orange protein has a role in phytoene synthase stabilization in sweetpotato
Source: Sci Rep. 2016 Sep 16;6:33563. doi: 10.1038/srep33563 (PMC5025653; doi:10.1038/srep33563)
Supplement: Supplementary Information [file srep33563-s1.pdf]

Supplementary Information

Title: Orange protein has a role in phytoene synthase stabilization in sweetpotato

Seyeon Park[1,2,‡] , Ho Soo Kim[1,‡], Young Jun Jung[3,4], Sun Ha Kim[1], Chang Yoon Ji[1,2], Zhi Wang[5], Jae Cheol Jeong[1,2], Haeng-Soon Lee[1,2], Sang Yeol Lee[3] and Sang-Soo Kwak[1,2,*]

[1]Plant Systems Engineering Research Center, Korea Research Institute of Bioscience and Biotechnology (KRIBB), 125 Gwahak-ro, Daejeon 34141, Korea. [2]Department of Green Chemistry and Environmental Biotechnology, Korea University of Science and Technology (UST), 217 Gajeong-ro, Daejeon 34113, Korea. [3]Division of Applied Life Science (BK21 Plus program), Gyeongsang National University, 501 Jinjudae-ro, Jinju 52828, Korea. [4]National Institute of Ecology, 1210 Geumgang-ro, Maseo-myeon, Seocheon-gun 33657, Korea. [5]Institute of Soil and Water Conservation, Chinese Academy of Science and Ministry of Water Resources, Northwest A & F University, Shaanxi 712100, China

‡ These authors contributed equally to this work.

*To whom correspondence should be addressed:

Dr. Sang-Soo Kwak

Plant Systems Engineering Research Center, Korea Research Institute of Bioscience and Biotechnology (KRIBB), Daejeon 34141, Republic of Korea.

Phone: +82-42-860-4432; Fax: +82-42-860-4489; E-mail: sskwak@kribb.re.kr

Methods

Cloning and preparation of recombinant proteins.

Full-length *IbOr* and truncated fragments were cloned by PCR from the pGWB11-*IbOr-Wt* plant expression vector[7], and *IbPSY* was cloned from sweetpotato. Full-length *IbPSY* sequence (GenBank Accession No. JX393305) was obtained using PCR with a pair of forward and reverse primers (Table S1) that were synthesized based on partial *IbPSY* sequence (GenBank Accession No. HQ828092) and allied species (*Ipomoea* sp. *Kenyan*, GenBank Accession No. AB499050.1). PCR and cloning were performed as described previously[7].

To construct GST-fused IbOr, full-length *IbOr* and truncated fragments (*IbOr-N*, *IbOr-C*, *IbOr-N1*, and *IbOr-N2*) and *IbPSY* were amplified using synthetic oligonucleotide primers including restriction enzyme sites (*IbOr* and *IbOr* fragments: *EcoRI* and *XhoI*; *IbPSY*: *BamHI* and *EcoRI*) (Table S1). The PCR products were cloned into the T-blunt vector (Solgent, Deajeon, Korea) and sequenced. *IbOr*, *IbOr* fragments, and *IbPSY* clones were digested with restriction enzymes and ligated into the pGEX-5X-1 vector (GE Healthcare, Uppsala, Sweden), which contained the IPTG-inducible *tac* promoter and an N-terminal GST tag, and then transformed into *E. coli* BL21 (DE3). The GST-fused proteins were induced by 1 mM IPTG at 15°C for 8 h and total membrane proteins were obtained. Membrane proteins were solubilized by 1% triton X-100 at 4°C for 1h. The GST-fused proteins were obtained using glutathione Sepharose (GE Healthcare, Uppsala, Sweden) and dialyzed against 50 mM Tris-HCl (pH 8.0) for use in biochemical analyses.

To construct His-fused IbPSY, *IbPSY* was cloned into a His-containing vector using Gateway cloning technology. *IbPSY* was cloned by PCR and amplified using synthetic oligonucleotide primers including *attB* sites and adapter primers (Table S1). The PCR and cloning were performed as described previously[7]. The PCR product was cloned into the pDONR207 vector (Invitrogen, Carlsbad, CA USA), moved to the pDEST17 vector (Invitrogen, Carlsbad, CA USA), which contained the *T7* promoter and N-terminal His

tag, and then transformed into *E. coli* BL21 (DE3). The His-fused IbPSY was induced by 1 mM IPTG at 15°C for 8 h and total membrane proteins were obtained. Membrane proteins were solubilized by 1% triton X-100 at 4°C for 1h. His-fused IbPSY was obtained using Ni-NTA (Qiagen, Hilden, Germany).

For laser scanning confocal microscopy analysis, *IbOr* and *IbPSY* were fused with GFP using Gateway cloning technology. pDONR207-*IbOr*[7] and pDONR207-*IbPSY* were subjected to site-specific recombination into pMDC83, which contained the cauliflower mosaic virus *35S* promoter and the C-terminal GFP protein[47].

For BiFC assay, *IbOr* and *IbPSY* were fused with Venus-C and Venus-N, respectively, using Gateway cloning technology. pDONR207-*IbOr* and pDONR207-*IbPSY* were subjected to site-specific recombination into pVyCE and pVyNE, which contained the cauliflower mosaic virus 35S promoter and the C-terminal or N-terminal of Venus protein (improved YFP derivative)[48].

For the LCI assay, *IbOr* and *IbPSY* were cloned by PCR and amplified using synthetic oligonucleotide primers including restriction enzyme sites (*IbOr*: *KpnI* and *BamHI*; *IbPSY*: *BamHI* and *SalI*) (Table S1). The PCR products were cloned into the T-blunt vector (Solgent, Daejeon, Korea) and sequenced. *IbOr* and *IbPSY* clones were digested with restriction enzymes and ligated into the pCAMBIA-NLuc and pCAMBIA-CLuc vectors[49].

For the Y2H assay, *IbOr-N*, *IbOr-C*, and *IbPSY* were cloned by PCR and amplified using synthetic oligonucleotide primers including restriction enzyme sites (*EcoRI* and *BamHI*) (Table S1). The PCR products were cloned into the T-blunt vector (Solgent, Daejeon, Korea) and sequenced. *IbOr-N* and *IbOr-C* clones were digested with restriction enzymes and ligated into the pGAD424 vector (Clontech, Mountain View, CA USA), which contained the *ADH1* promoter and N-terminal GAL4-activation domain. The *IbPSY* clone was digested with restriction enzymes and ligated into the pAS2-1 vector (Clontech, Mountain View, CA USA), which contained the *ADH1* promoter and N-terminal GAL4-binding domain.

To evaluate IbOr holdase chaperone activity *in planta*, *GUS* was amplified using the synthetic oligonucleotide primers including restriction enzyme sites (*BamHI* and *SalI*) (Table S1) from the

pENTR™ GUS vector (Invitrogen, Carlsbad, CA USA). The PCR product was cloned into the T-blunt vector (Solgent, Daejeon, Korea) and sequenced. *GUS* clones were digested with restriction enzymes and ligated into the pCAMBIA1300-multi vector, which contained the cauliflower mosaic virus *35S* promoter.

Oligomerization status of IbOr under heat-shock conditions.

The oligomerization status of IbOr was analyzed using GST:IbOr incubated for 30 min at 25, 30, 35, 40, 45, and 50°C in water. Native PAGE was performed to detect protein oligomerization.

Thermostability test.

Equal amounts of GST and GST:IbOr proteins were incubated for 30 min at 25, 40, 50, 60, and 70°C in 50 mM Tris-HCl, pH 8.0, followed by centrifugation to remove aggregated proteins. The proteins were analyzed by SDS-PAGE.

Quantitative Real-time PCR.

Three-week-old sweetpotato plants and sweetpotato calli 10 d after subculture were subjected to 38°C for 1, 3, 6, 12, and 24 h. The third or fourth leaves, stems, fibrous roots, and sweetpotato calli were used for RNA extraction. Total RNA was extracted from the samples using RNAiso Plus regent (Takara, Otsu, Japan), followed by treatment with RNase-free DNase I (Takara, Otsu, Japan) to remove genomic DNA contamination. First-strand cDNA synthesis was performed from total RNA (1 μg) using TOPscript™ RT DryMIX (*dT18*) (Enzynomics, Daejeon, Korea) according to the manufacturer's instructions. The primer pair for *IbOr* (Accession no. HQ828087) was designed with the Primer3Plus program (http://www.bioinformatics.nl/cgi-bin/primer3plus/primer3plus.cgi/). The gene-specific forward and reverse primers (Table S1) were chosen so that the amplicon size was 77 bp. Real-time PCR analysis was performed in 96-well plates with a CFX real-time PCR system and CFX system software (Bio-Rad, Hercules, CA USA) using the EvaGreen-based PCR assay as described previously[50].

Firefly luciferase complementation imaging (LCI) assay.

Constructs were transformed into *Agrobacterium tumefaciens* GV3101. *Agrobacterium*-mediated transient expression for LCI assay, CCD imaging, and LUC activity measurement were performed as described previously[49].

Pull-down assay.

His-IbPSY was incubated with GST or GST-IbOr for 4 h at 4°C, and then mixed with glutathione Sepharose beads for 12 h at 4°C in 1 ml of binding buffer (50 mM Tris-HCl, pH 7.5, 250 mM NaCl, 5 mM EDTA, 0.1% Tween 20, 1 mM DTT, and protease inhibitor cocktail). Beads were washed and resuspended in 20 μl of 4×SDS sample buffer. Proteins were detected by immunoblotting with anti-His.

Yeast two-hybrid (Y2H) assay.

Constructs were transformed into yeast strain PJ69-4A. Transformant growth was monitored on synthetic complete medium lacking Trp and Leu, and in the presence or absence of His. Three independent transformants were tested.

Total chlorophyll content measurement.

Total chlorophyll content was measured using 0.1 g (fresh weight) of leaf tissue that was quickly frozen in liquid nitrogen and then extracted with 1 ml of 80% acetone. Samples were briefly centrifuged, and supernatants were analyzed using a spectrophotometer as described previously[51].

Ion leakage analysis.

Ion leakage due to oxidative stress was examined by treating rosette leaves of *At*-EV and *At*-OX with 5 mM H_2O_2 or 10 μM methyl viologen for 24 h at 22°C. Ion leakage was measured with the ion conductivity meter model 455C (Istek, Seoul, Korea) during a 0–24 h period as described previously[40].

References

47. Curtis, M. D. & Grossniklaus, U. A gateway cloning vector set for high-throughput functional analysis of genes in planta. *Plant Physiology* **133,** 462-469 (2003).

48. Gehl, C., Waadt, R., Kudla, J., Mendel, R. R. & Hänsch, R. New GATEWAY vectors for high throughput analyses of protein–protein interactions by bimolecular fluorescence complementation. *Molecular plant* **2,** 1051-1058 (2009).

49. Chen, H. *et al.* Firefly luciferase complementation imaging assay for protein-protein interactions in plants. *Plant Physiology* **146,** 368-376 (2008).

50. Park, S. C. *et al.* Stable internal reference genes for the normalization of real-time PCR in different sweetpotato cultivars subjected to abiotic stress conditions. *PLoS ONE* **7,** e51502 (2012).

51. Arnon, D. I. Copper enzymes in isolated chloroplasts. Polyphenoloxidase in beta vulgaris. *Plant Physiology* **24,** 1-15 (1949).

Table S1. List of primer pairs used in this study.

Name, purpose	Forward (5'-3')	Reverse (5'-3')
***IbOr*, qPCR**	TGGAAGGCTCAAATCCAGAG	CGACGGATGAAGAAAAGGAG
***IbPSY*, CDS cloning**	ATGTCAAGTGTCTTGCTGTG	TCAAGCTTTTGCCAGAGG
***IbOr*, GST-fusion**	GAATTCATGGTATATTCAGGTAGAATCTTGTCG	CTCGAGTTAATCAAATGGGTCAATTCGTGG
***IbOr-N*, GST-fusion**	GAATTCATGGTATATTCAGGTAGAATCTTGTCG	CTCGAGCTCCTGCTGTTTCACATTGTT
***IbOr-C*, GST-fusion**	GAATTCCATAAGAGGTGCAAGTACTGTTTAGG	CTCGAGTTAATCAAATGGGTCAATTCGTGG
***IbOr-N1*, GST-fusion**	GAATTCATGGTATATTCAGGTAGAATCTTGTCG	CTCGAGCCCAAGCTCAGCATTCTTAATT
***IbOr-N2*, GST-fusion**	GAATTCAATCTTAATGAAAAGCAAGAAAATAAACTTCCGA	CTCGAGCTCCTGCTGTTTCACATTGTT
***IbPSY*, GST-fusion**	GGATCCTTATGTCAAGTGTCTTGCTGTG	GAATTCTCAAGCTTTTGCCAGAGGGGAA
***IbPSY*, Gateway**	AAAAAGCAGGCTTAATGTCAAGTGTCTTGCTG	AGAAAGCTGGGTGAGCTTTTGCCAGAGGGGA
**Adapter, Gateway**	GGGGACAAGTTTGTACAAAAAAGCAGGCT	GGGGACCACTTTGTACAAGAAAGCTGGGT
***IbOr*, LCI assay**	GGTACCATGGTATATTCAGGTAGAATC	GGATCCATCAAATGGGTCAATTCGTGG
***IbPSY*, LCI assay**	GGATCCAATGTCAAGTGTCTTGCTGTGG	GTCGACTCAAGCTTTTGCCAGAGGGGA
***IbOr-N*, Y2H assay**	GAATTCATGGTATATTCAGGTAGAATC	GGATCCCTCCTGCTGTTTCACATTGTT
***IbOr-C*, Y2H assay**	GAATTCCATAAGAGGTGCAAGTACTGTTTAGG	GGATCCTTAATCAAATGGGTCAATTCGTGG
***IbPSY*, Y2H assay**	GAATTCATGTCAAGTGTCTTGCTGTGGG	GGATCCTCAAGCTTTTGCCAGAGG
***GUS*, Chaperone activity**	GGATCCATGTTACGTCCTGTAGAAAC	GTCGACTTATTGTTTGCCTCCCTGCT

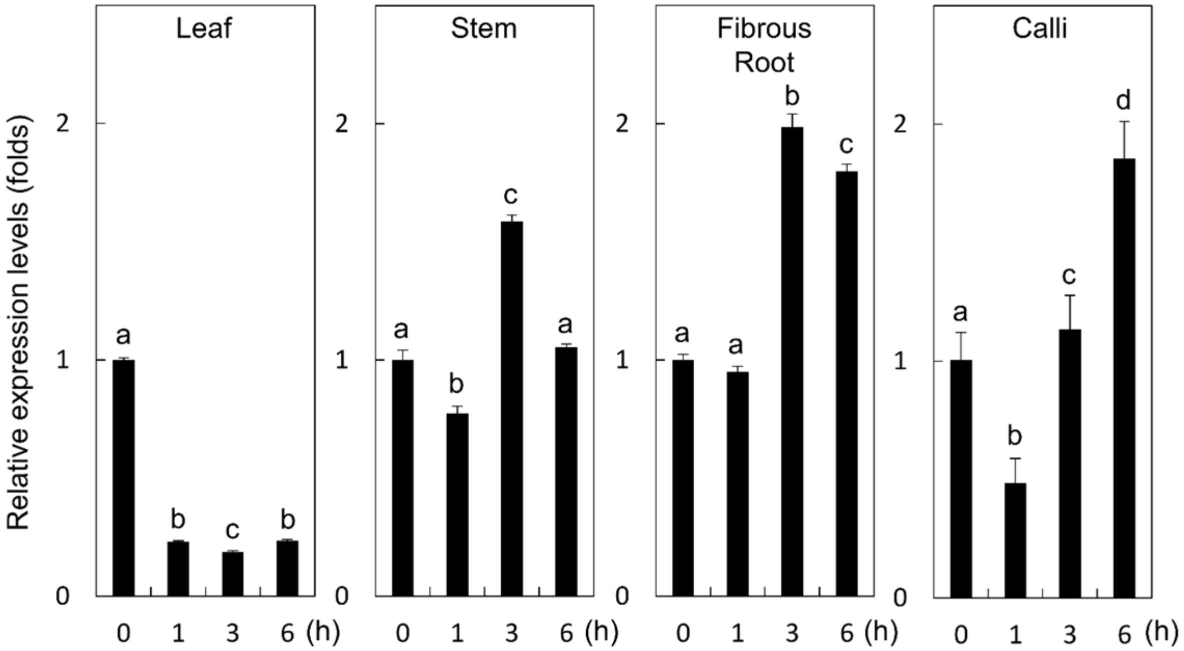

Supplementary Fig. S1 | *IbOr* expression profiles in different sweetpotato tissues under heat stress conditions. *IbOr* transcript expression levels were measured by qRT-PCR analysis under heat stress at 38°C. Results are the means ± SD from three biological replicates. Different letters indicate statistically significant differences ($p < 0.05$).

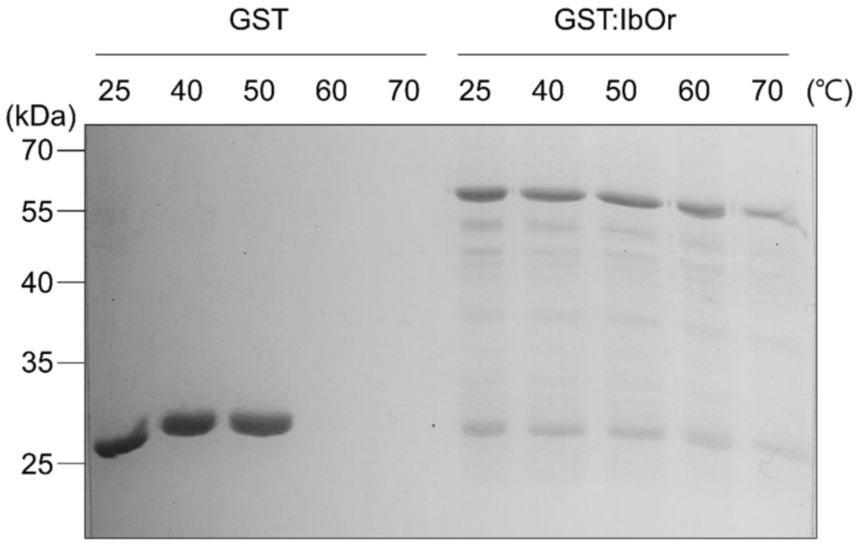

Supplementary Fig. S2 | IbOr thermostability *in vitro*. Purified GST and GST:IbOr were incubated at the indicated temperatures for 30 min, and then analyzed on a silver-stained 12% SDS-PAGE gel.

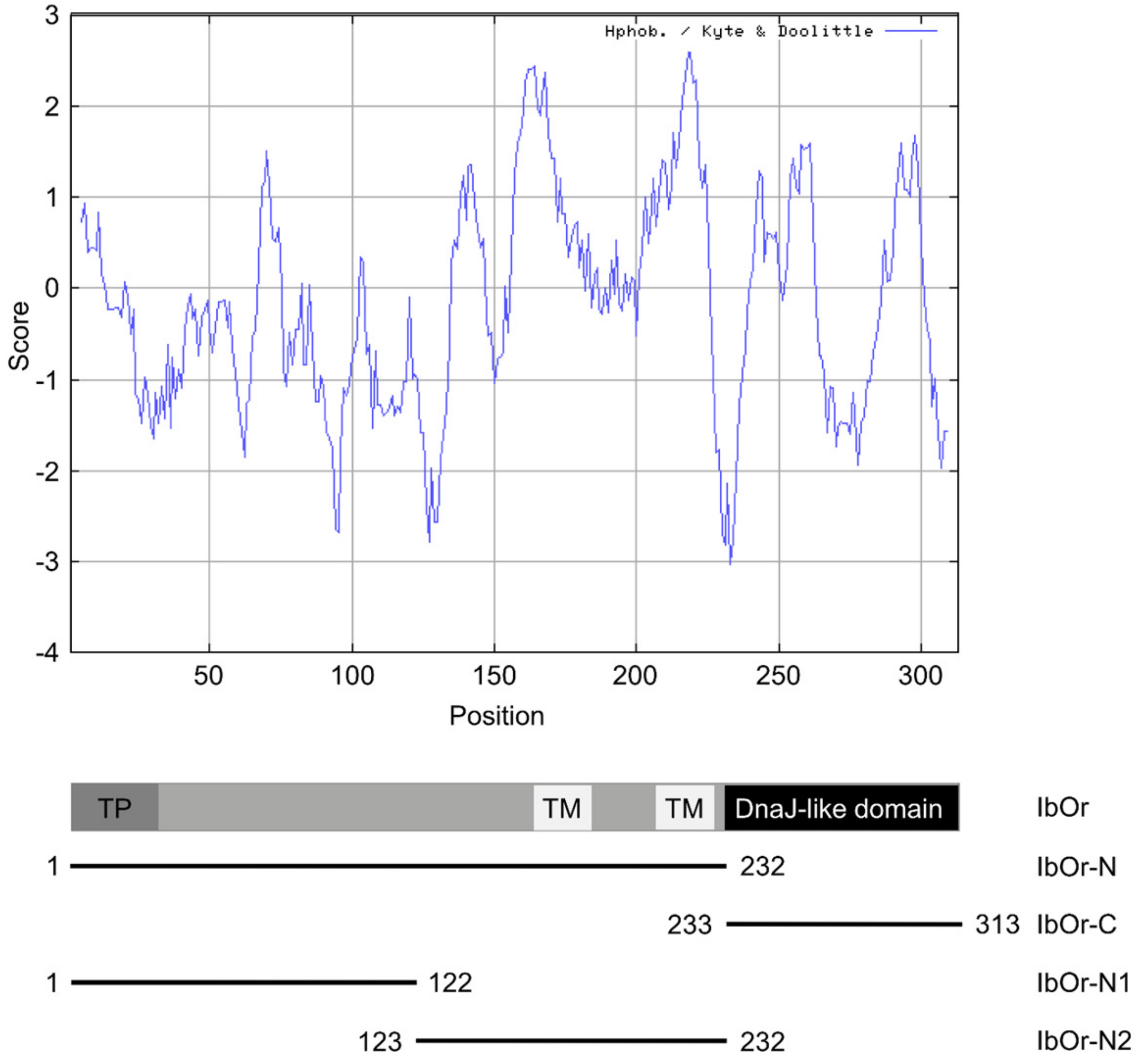

Supplementary Fig. S3 | Hydrophobicity prediction for IbOr. The Kyte & Doolittle hydrophobicity profile of IbOr is shown (top panel). Schematic of full-length IbOr and truncated fragments (oriented with respect to the positions in the hydrophobicity curve *x*-axis) (bottom panel).

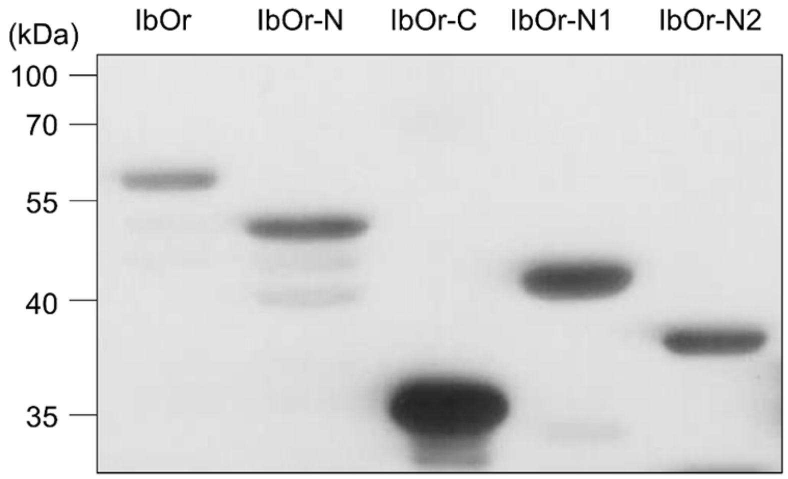

Supplementary Fig. S4 | Anti-GST immunoblotting of GST-fused IbOr and its truncated fragments. GST-fused IbOr and its truncated fragments were analyzed on 12% SDS-PAGE by immunoblotting with anti-GST antibody.

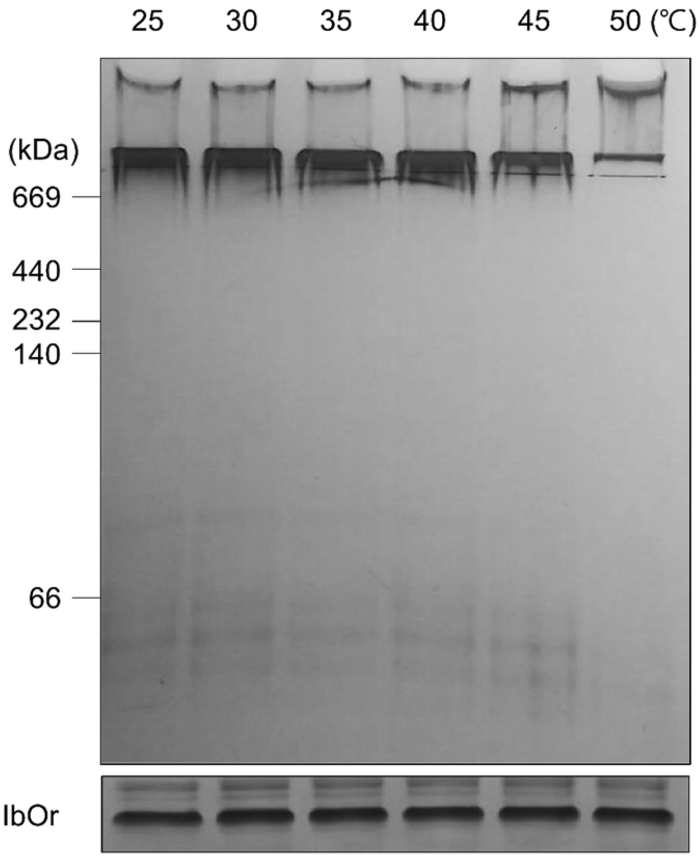

Supplementary Fig. S5 | Heat-shock-dependent change of IbOr oligomeric status *in vitro*. IbOr was subjected to the indicated temperatures for 30 min, and changes in IbOr oligomeric status were analyzed on a silver- stained 10% native PAGE gel (top panel) or a 12% SDS-PAGE gel (bottom panel).

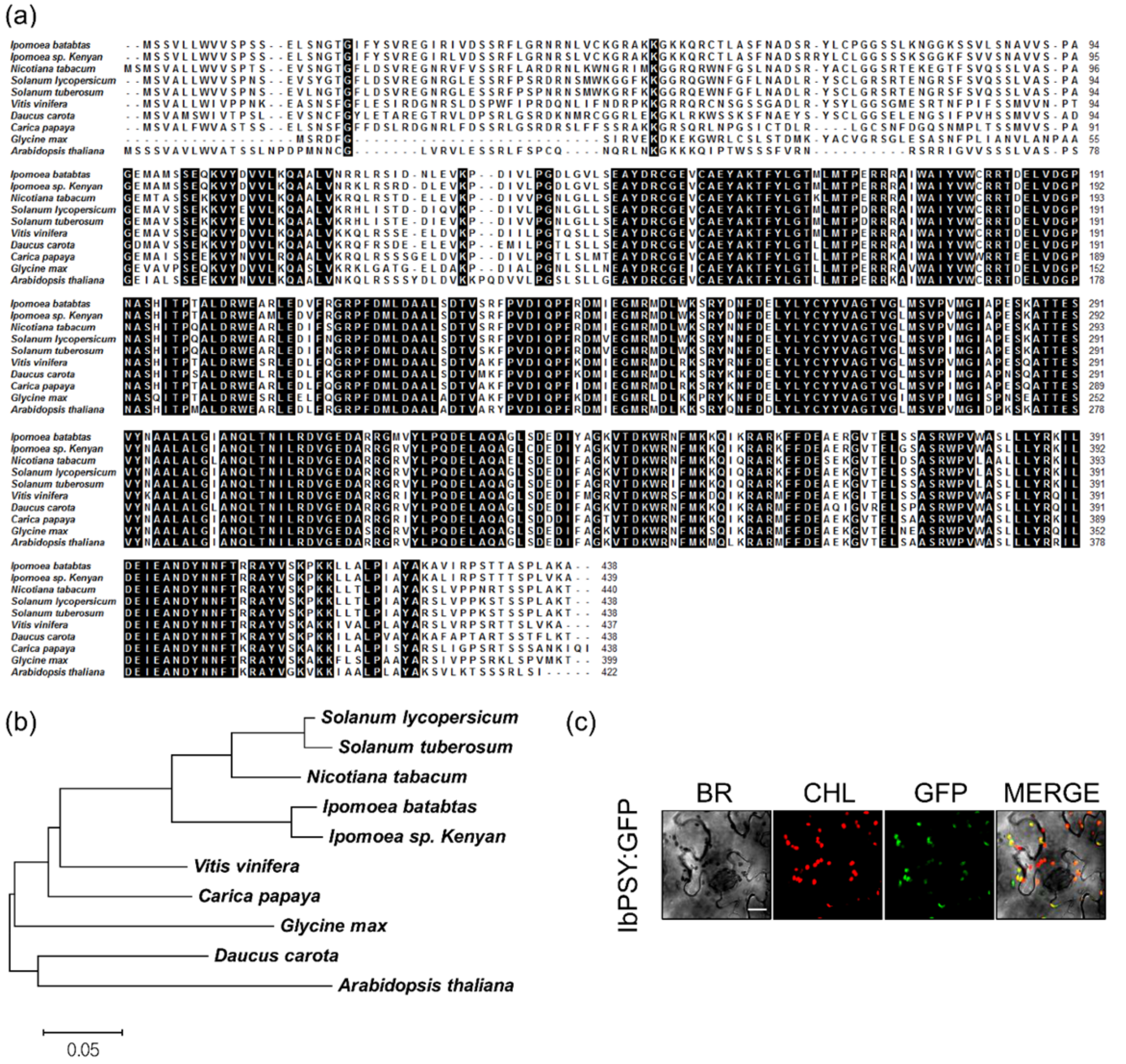

Supplementary Fig. S6 | Characterization and structural analysis of *IbPSY*. (a) Comparison of deduced amino acid sequences of IbPSY and other PSY proteins. The GenBank accession numbers for the PSY subfamily are as follows: *Ipomoea batatas* (Accession no. JX393305), *Ipomoea* sp. *Kenyan* (Accession no. BAI47572.1), *Nicotiana tabacum* (Accession no. AHA58684.1), *Solanum lycopersicum* (Accession no. NP_001234671.1), *Solanum tuberosum* (Accession no. XP_006348183.1), *Vitis vinifera* (Accession no. XP_002271575.1), *Daucus*

carota (Accession no. ABB52068.1), *Carica papaya* (Accession no. ABG72805.1), *Glycine max* (Accession no. NP_001242405.1), and *Arabidopsis thaliana* (Accession no. NP_001031895.1). (b) Phylogenetic analysis of IbPSY and other PSY proteins. Amino acid sequence alignment was performed using the ClustalW multiple sequence alignment program MEGA6. (c) Subcellular localization of IbPSY:GFP. GFP-fused IbPSY was transiently expressed in *N. benthamiana* leaves by agroinfiltration and observed by confocal laser scanning microscopy. Green fluorescence of IbPSY:GFP was detected in chloroplasts. BR, bright field microscopy images; CHL, chlorophyll autofluorescence images; GFP, GFP fluorescence images; MERGE, overlay images of bright field, chlorophyll, and GFP fluorescence. Scale bar = 20 μm.

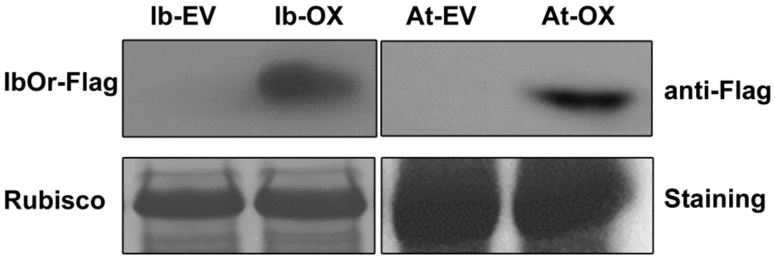

Supplementary Fig. S7 | IbOr expression levels in sweetpotato and *Arabidopsis* transgenic plants. EV and OX total proteins were analyzed on 12% SDS-PAGE by immunoblotting with anti-FLAG antibody. Rubisco was used as a loading control.

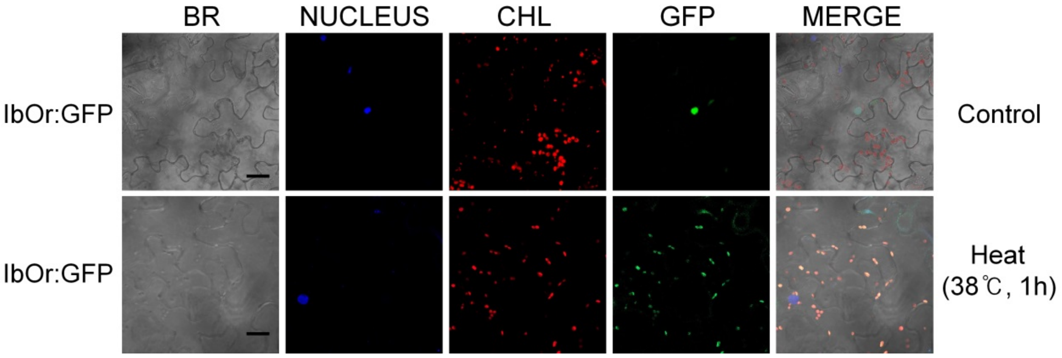

Supplementary Fig. S8 | Subcellular localization of IbOr:GFP fusion protein in *N. benthamiana* under normal and heat stress conditions. IbOr:GFP was transiently expressed in *N. benthamiana* leaves by agroinfiltration and observed by confocal laser scanning microscopy. After agroinfiltration, the infiltrated leaves were detached and cut into small squares, fixed, and stained with DAPI to label nuclei. Green fluorescence of IbOr:GFP was mainly detected in the nucleus under normal conditions, but was detected only in chloroplasts under heat stress conditions. BR, bright field microscopy images; NUCLEUS, DAPI fluorescence images; CHL, chlorophyll autofluorescence images; GFP, GFP fluorescence images; MERGE, overlay images of bright field, nucleus, chlorophyll, and GFP fluorescence images. Scale bar = 20 μm.
